# Comparative Study on Single-Molecule Junctions of Alkane- and Benzene-Based Molecules with Carboxylic Acid/Aldehyde as the Anchoring Groups

**DOI:** 10.1186/s11671-016-1596-1

**Published:** 2016-08-26

**Authors:** Fang Chen, Lin-Lu Peng, Ze-Wen Hong, Jin-Chuan Mao, Ju-Fang Zheng, Yong Shao, Zhen-Jiang Niu, Xiao-Shun Zhou

**Affiliations:** Key Laboratory of the Ministry of Education for Advanced Catalysis Materials, Institute of Physical Chemistry, Zhejiang Normal University, Jinhua, Zhejiang 321004 China

**Keywords:** Single-molecule junctions, ECSTM-BJ, Junction formation probability, Carboxylic acid, Aldehyde

## Abstract

We have measured the alkane and benzene-based molecules with aldehyde and carboxylic acid as anchoring groups by using the electrochemical jump-to-contact scanning tunneling microscopy break junction (ECSTM-BJ) approach. The results show that molecule with benzene backbone has better peak shape and intensity than those with alkane backbone. Typically, high junction formation probability for same anchoring group (aldehyde and carboxylic acid) with benzene backbone is found, which contributes to the stronger attractive interaction between Cu and molecules with benzene backbone. The present work shows the import role of backbone in junction, which can guide the design molecule to form effective junction for studying molecular electronics.

## Background

In recent years, single-molecule junctions have attracted wide attention because of its potential application in nano-electronic and molecular electronic device [1–10]. At present stage, it is important to fully understand the electron transport of single-molecule junctions and its influence factors [11]. Many factors can affect the conductance of single-molecule junctions, such as anchoring group, molecule structure, the contact configuration between molecule and electrode, and temperature [5, 7, 12–17]. Among them, anchoring group is very important in forming the molecular junction, and it was found that different anchoring groups have different junction formation probabilities [14, 18–20]. However, another interesting question is still unclear that how molecular structure would influence the junction formation probability for same anchoring group.

**Fig. 1:**
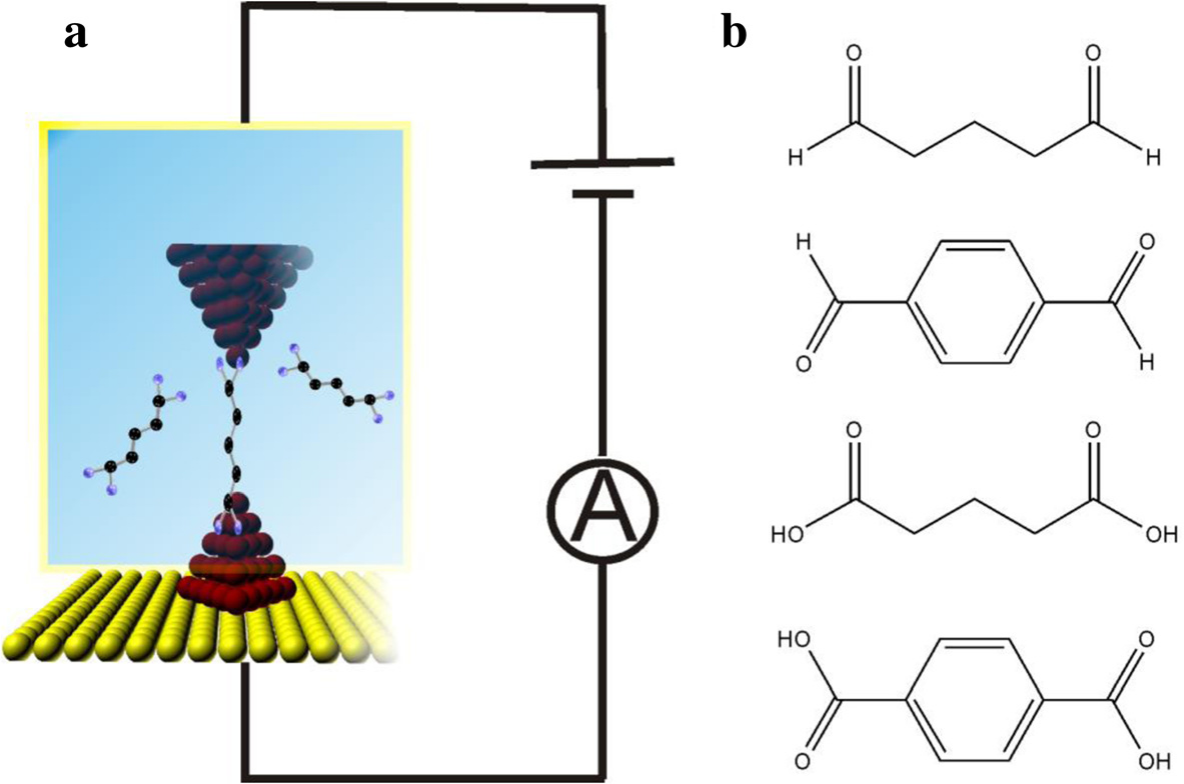
The schematic diagram of ECSTM-BJ and molecular structure. **a** Schematic diagram of electrochemical jump-to-contact scanning tunneling microscopy break junction (ECSTM-BJ) approach in solution containing target molecule. **b** Molecular structures of glutaraldehyde, 1,4-benzenedicarboxaldehyde, pentanedioic acid and 1,4-benzenedicarboxylic acid

In this work, we will focus on the junction formation probability of same anchoring group with different molecular structures (saturated and conjugated structure) by using electrochemical jump-to-contact scanning tunneling microscopy break junction (ECSTM-BJ) approach (Fig. 1a) [21, 22]. Aldehyde and carboxylic acid anchoring groups binding to Cu electrode are used in the current study, for they have been demonstrated to form effective junctions [23–25]. We use 1,4-benzenedicarboxaldehyde, glutaraldehyde, 1,4-benzenedicarboxylic acid and pentanedioic acid as target molecules (Fig. 1b) to study the influence of different structures on the junction formation probability. Those molecules have different backbones with saturated (alkane) or conjugated (benzene) structure.

## Methods

Na_2_SO_4_ (99.995 %) and CuSO_4_ (99.999 %) were purchased from Alfa-Aesar, while petanedioic acid, 1,4-benzenedicarboxylic acid, glutaraldehyde, and 1,4-benzenedicarboxaldehyde were purchased from Sigma-Aldrich. Ultrapure water (≥18.2 MΩ cm) was used for preparing aqueous solutions. Naturally formed Au(111) was used as the substrate, and cut Pt-Ir STM tip was covered with thermosetting glue to reduce the electrochemical current. Meantime, Pt and Cu wires were used as the counter and reference electrodes, respectively.

The conductance measurement was performed by ECSTM-BJ approach on the modified Nanoscope IIIa STM (Veeco, USA); especially, preamplifier with four-out linear current-to-voltage converters was used [23]. The experiment was carried out in aqueous solution containing saturated target molecule + 1 mM CuSO_4_ + 50 mM Na_2_SO_4_ as following: Firstly, the tip potential was set at −5 mV to allow the bulk deposition of Cu. Secondly, after applying the pulse voltage on z-piezo, the deposited Cu on the tip would transfer to the substrate and build a metallic contact due to the tip closed to the surface. Thirdly, Cu atomic wire could be formed during the separation of tip and substrate with 20 nm/s, and then, molecule could simultaneously bridge to the both electrodes upon the breaking of metal atomic wire. The tip current vs. distance curves were recorded with sampling frequency of 20 kHz. More detailed procedure can be seen in our previously reports [22, 23].

## Results and Discussion

### Comparative Study on Single-Molecule Conductance of Glutaraldehyde and 1,4-Benzenedicarboxaldehyde with Cu Electrode

**Fig. 2:**
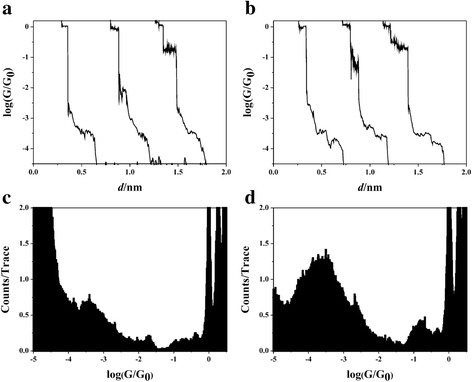
Single-molecule conductance histograms of pentanedioic acid and glutaraldehyde. A log-scale conductance curves of **a** Cu-glutaraldehyde-Cu junctions and **b** Cu-1,4-benzenedicarboxaldehyde-Cu junctions. Log-scale conductance histogram of **c** Cu-glutaraldehyde-Cu junctions and **d** Cu-terephthalaldehyde-Cu junctions

We firstly measure the conductance of Cu-glutaraldehyde-Cu junction. Conductance curves with obvious step can be seen in Fig. 2a and then were treated by logarithm and binning to construct the histogram (Fig. 2c). A peak at 10^−3.45^ G_0_ (27 nS) is found in the Fig. 2c. Comparing with glutaraldehyde, pronounced peak at 10^−3.6^ G_0_ (19 nS) is found for 1,4-benzenedicarboxaldehyde (Fig. 2b, d), and this value is consistent with our previously report [23]. Obviously, the peak intensity of 1,4-benzenedicarboxaldehyde is higher than that of glutaraldehyde. And the different intensity of peaks between glutaraldehyde and 1,4-benzenedicarboxaldehyde in the histograms may show internal property of benzene and alkane backbone.

**Fig. 3:**
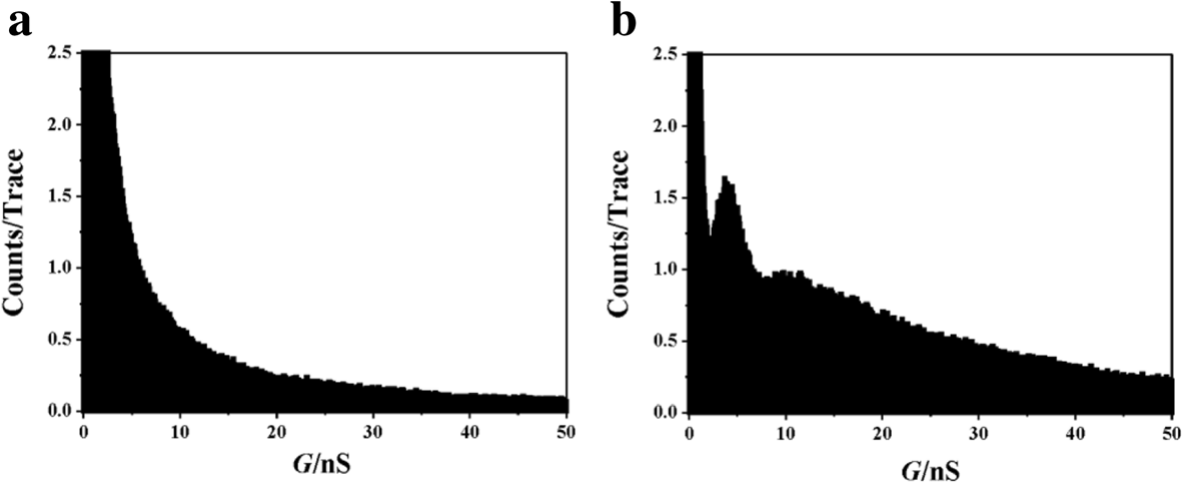
Comparison of the linear-scale conductance histograms of glutaraldehyde and 1,4-benzenedicarboxaldehyde. **a** The linear-scale conductance histogram of **a** Cu-glutaraldehyde-Cu junctions and **b** Cu-1,4-benzenedicarboxaldehyde-Cu junctions

Then, we also construct histograms by using linear bin-size. Obvious difference is again observed for those molecules in Fig. 3. While rather weak peak can be found for petanedioic acid (Fig. 3a), pronounced peak is shown for 1,4-benzenedicarboxaldehyde (Fig. 3b). Conductance values of 4 and 11 nS are found for 1,4-benzenedicarboxaldehyde, which is different from the conductance value shown in log-scale histogram (Fig. 2d). This is caused by the different statistical methods between linear and logarithm bin-size, and different molecule-electrode configurations are shown in those histograms [23]. As our previously report, we can also obtain all conductance values of 4, 11, and 20 nS using data selection with linear bin-size [23]. However, the linear-bin histograms show even large difference of intensity between molecules with benzene and alkane backbone.

Return back to the difference histograms between benzene and alkane backbone, the weak peak can be caused by the less probability in the forming the molecular junctions. Usually, the junction formation probability can be analyzed by stretched distance distribution [26, 27] or counting the number of curves with step [28, 29] as previously reports. Here, we manually analyze the opportunity of step (typically, the curve with step length longer than 0.05 nm) in conductance curves showing step value smaller than 10^−2^ G_0_, it is found that step opportunity is around 40 % in forming junction of 1,4-benzenedicarboxaldehyde, while around 22 % for glutaraldehyde. From above, we can conclude that the anchoring group of aldehyde with benzene backbone has high junction formation probability than that with alkane backbone connecting with Cu electrode, and we will discuss it later.

### Comparative Study on Single-Molecule Conductance of Pentanedioic Acid and 1,4-Benzenedicarboxylic Acid with Cu Electrode

**Fig. 4:**
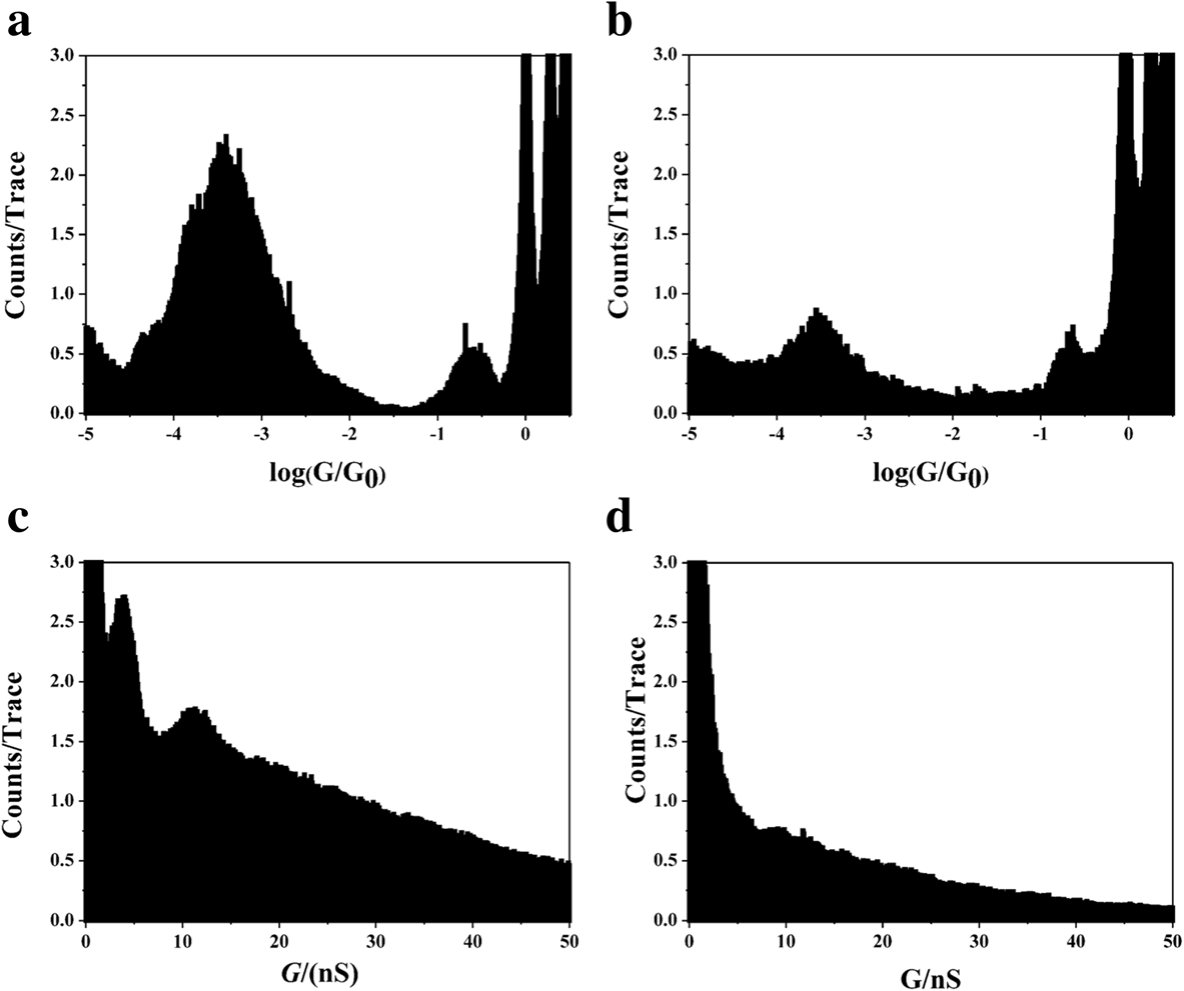
Comparison of linear-scale and log-scale conductance histogram of 1,4-benzenedicarboxylic acid and petanedioic acid. The log-scale conductance histograms of **a** Cu-1,4-benzenedicarboxylic acid-Cu junctions and **b** Cu-petanedioic acid-Cu junctions. The linear-scale conductance histograms of **c** Cu-1,4-benzenedicarboxylic acid-Cu junctions and **d** Cu-petanedioic acid-Cu junctions

In order to prove the role of backbone in forming molecular junction, we also use carboxylic acid as the anchoring group to comparing difference between petanedioic acid and 1,4-benzenedicarboxylic acid. As shown in Fig. 4, similar behavior is also found that 1,4-benzenedicarboxylic acid shows more pronounced peak comparing with petanedioic acid. Again, different conductance values are found in different statistical method between linear and log bin-size. According to Fig. 4, the junction formation probability of 1,4-benzenedicarboxylic acid is higher than that of petanedioic acid in both linear-scale and log-scale statistical histograms. We found that the step opportunity of 1,4-benzenedicarboxylic acid and petanedioic acid is around 51 % and 33 %, respectively, which illustrates the similar results as the 1,4-benzenedicarboxaldehyde and glutaraldehyde. However, molecules with carboxylic acid have larger junction formation probability than those with aldehyde anchoring group; this may be caused by that carboxylic acid can also bind to the Cu through carboxylate form with two O atoms binding to the electrode, while only one O atom can bind to the electrode for aldehyde group.

### The Role of Backbone in Forming Molecular Junction

According to above results, those molecules with benzene backbone have higher junction formation probabilities than those with alkane backbone connecting with Cu electrode, which should be caused by the stronger interaction between anchoring group and Cu in 1,4-benzenedicarboxaldehyde and 1,4-benzenedicarboxylic acid.

Taking carboxylic acid as example, carboxylic acid binds to the Cu electrode through carboxylate group [30]. It was reported that the bond length of Cu-O for benzene system is shorter than that of alkane system, which may reveal that benzene-based molecule and Cu system has stronger attractive interaction than that of alkane-based molecule and Cu system [30, 31]. This can explain our result that the junction formation probability of 1,4-benzenedicarboxylic acid is higher than that of petanedioic acid. We deduce the similar situation for molecules with aldehyde anchoring group, since similar Cu–O bond is formed in the junctions [23]. The current work shows the import role of backbone in forming molecular junctions and may help the design of molecule in studying the electron transport of single-molecule junction.

## Conclusions

In this work, we have measured the single molecular junction conductance of molecules with aldehyde and carboxylic acid anchoring groups. It has been found that the structure of backbone can influence the junction formation probability for same anchoring group (aldehyde and carboxylic acid), which contributes to the stronger attractive interaction between Cu and molecules with benzene backbone. Those results can guide the design molecule to form effective junction for studying molecular electronics.
